# Valorization of a Lanthanum-Modified Natural Feedstock for Phosphorus Recovery from Aqueous Solutions: Static and Dynamic Investigations

**DOI:** 10.3390/ma18143383

**Published:** 2025-07-18

**Authors:** Hamed Al-Nadabi, Salah Jellali, Wissem Hamdi, Ahmed Al-Raeesi, Fatma Al-Muqaimi, Afrah Al-Tamimi, Ahmed Al-Sidairi, Ahlam Al-Hanai, Waleed Al-Busaidi, Khalifa Al-Zeidi, Malik Al-Wardy, Mejdi Jeguirim

**Affiliations:** 1 Center for Environmental Studies and Research, Sultan Qaboos University, Al-Khoud 123, Muscat P.O. Box 17, Oman; hamed@squ.edu.om (H.A.-N.); aalraeesi@squ.edu.om (A.A.-R.); fatmaalmuqimi3@gmail.com (F.A.-M.); achemtamimi@gmail.com (A.A.-T.); sidairi@squ.edu.om (A.A.-S.); aalhinai@squ.edu.om (A.A.-H.); alzeidi@squ.edu.om (K.A.-Z.); mwardy@squ.edu.om (M.A.-W.); 2 Higher Institute of the Sciences and Techniques of Waters, University of Gabes, Gabes 6033, Tunisia; wissemhemdi@yahoo.fr; 3 College of Agricultural and Marine Sciences, Sultan Qaboos University, Al-Khoud 123, Muscat P.O. Box 17, Oman; waleedm@squ.edu.om; 4 The Institute of Materials Science of Mulhouse (IS2M), University of Haute Alsace, Centre National de la Recherche Scientifque, Unité Mixte de Recherche 7361, F-68100 Mulhouse, France; mejdi.jeguirim@uha.fr

**Keywords:** phosphorus, magnetite, batch, CSTR, adsorption mechanisms

## Abstract

This work investigates, for the first time, the application of a modified natural magnetite material with 35% of lanthanum for phosphorus (P) recovery from synthetic and actual wastewater under both static (batch) and dynamic (continuous stirred tank reactor (CSTR)) conditions. The characterization results showed that the natural feedstock mainly comprises magnetite and kaolinite. Moreover, the lanthanum-modified magnetite (La-MM) exhibited more enhanced textural, structural, and surface chemistry properties than the natural feedstock. In particular, its surface area (82.7 m^2^ g^−1^) and total pore volume (0.160 cm^3^ g^−1^) were higher by 86.6% and 255.5%, respectively. The La-MM efficiently recovered P in batch mode under diverse experimental settings with an adsorption capacity of 50.7 mg g^−1^, which is significantly greater than that of various engineered materials. It also maintained high efficiency even when used for the treatment of actual wastewater, with an adsorption capacity of 47.3 mg g^−1^. In CSTR mode, the amount of P recovered from synthetic solutions and real wastewater decreased to 33.8 and 10.2 mg g^−1^, respectively, due to the limited contact time. The phosphorus recovery process involves mainly electrostatic attraction over a wide pH interval, complexation, and precipitation as lanthanum phosphates. This investigation indicates that lanthanum-modified natural feedstocks from magnetite deposits can be regarded as promising materials for P recovery from aqueous solutions.

## 1. Introduction

Phosphorus (P) is a vital element for all forms of life. It is usually extracted from non-renewable natural reserves. Some alarming reports have shown that the P reserves in several regions of the world are expected to decrease significantly during the next decades [1]. In parallel, approximately 3.0 million tons of phosphorus (P) are discharged into natural environments from wastewater [2]. This quantity represents almost 6.8% of the global P demand as a fertilizing macronutrient in agriculture and is expected to increase significantly during the coming decades [2]. Along with nitrogen, discharged P can seriously affect the quality of water bodies and harm their fauna and flora through the process of eutrophication [3]. Therefore, it is imperative to find suitable and cost-effective solutions for P recovery from effluents for subsequent reuse in agriculture as an alternative to synthetic P-based fertilizers derived from non-renewable reserves [4].

Nowadays, P recovery from effluents includes mainly biological accumulation, chemical precipitation, crystallization as struvite, and adsorption onto raw and engineered materials [5,6]. The latter is regarded as one of the most efficient and environmentally friendly technologies [7,8]. Moreover, it is a low-cost, practical, and simple-to-use method [9]. Over the past decade, various organic-based adsorbents such as activated carbon and biochar [10,11], as well as mineral-based materials including calcium- and/or magnesium-rich byproducts [8], zeolite [12], and magnetite-based materials [13], have been tested for effective P recovery from effluents. The use of magnetite offers key benefits such as availability, affordability, and environmental-friendliness. Nevertheless, due to its relatively poor physico-chemical properties, raw magnetite-based materials usually exhibit low P adsorption capacities [14,15]. Therefore, various modification methods have been tested for the enhancement of these characteristics and subsequently for the improvement of their ability to recover P from effluents [13].

The modification of magnetite-based materials with metal salts such as zirconium, zinc, magnesium, and lanthanum has been pointed out as an efficient and practical method [16,17,18,19]. Lanthanum (La) is usually preferred owing to its low cost, stability, eco-friendliness, and efficiency [20]. Depending on the experimental conditions, the P adsorption capacity of La-modified magnetite has been found to range between 7 to 50 mg g^−1^ [20,21]. However, a much larger value (>250 mg g^−1^) was observed for a La-modified magnetite with an exceptionally high lanthanum–magnetite percentage (128%) [15]. Moreover, most of the published studies have been conducted with synthetic magnetite prepared through ferric chloride (FeCl_3_) and ferrous chloride (FeCl_2_) co-precipitation at high alkaline pH values (greater than 10). This method is costly, unsustainable, and environmentally unfriendly [22]. Therefore, modifying samples collected from natural magnetite deposits with lanthanum and valorizing them, first for P recovery from effluents and subsequently as slow-release fertilizers in agriculture, can significantly boost sustainability and the circular economy [13,23,24].

The pre-treatment of magnetite with acidic solutions allows for the material to be coated with ferrihydrite nanoparticles (Fe_10_O_14_(OH)_2_), thereby enhancing its textural and surface chemistry properties [25]. In addition, lanthanum modification of this coated natural magnetite was found to improve its physico-chemical properties and also to further enhance P recovery efficiency from aqueous solutions [23]. However, most of the preceding studies were conducted under static (batch) conditions. Although such assays provide useful insights into the effects of specific experimental factors, they do not allow for adequate design of full-scale setups [26]. Therefore, moving to dynamic experiments using laboratory columns or continuous stirred tank reactors (CSTRs) has been identified as an essential step [24,26,27]. Indeed, contrarily to batch assays, dynamic experiments allow a continuous renewal of the effluent entering the treatment system, which is closer to real conditions [28]. Then, the obtained dynamic results can be used for an easy and adequate design of full-scale treatment settings [29,30]. In addition, the majority of previous laboratory investigations used synthetic solutions, which oversimplified the complex situations encountered in real-world scenarios. Therefore, testing real wastewater is highly recommended to adequately address the complexity of such media, which usually contain a diverse array of ions rather than just specific pollutants (i.e., P) [28,31].

In our recent study on P recovery with a La-modified natural Omani feedstock, collected from a magnetite deposit, we showed that P recovery efficiency increased from 12.4, to 24.3, and then to 34.5 mg g^−1^ when the La–magnetite percentage was raised from 0%, to 5%, and then to 15% [21]. Furthermore, P recovery efficiency was found to be highly dependent on the batch experimental conditions, especially the initial pH of effluent, the adsorbent dose, and the aqueous P concentration [21]. This preliminary study has provided valuable insights into P recovery in batch mode. However, the effect of using a higher La–magnetite percentage, real wastewater instead of synthetic solutions, and dynamic devices (i.e., CSTR) on P recovery performance is still lacking. Hence, in the current work, we first synthesized a La-modified natural magnetite sample at a higher La–feedstock percentage (35%) and then applied it for P recovery from aqueous solutions under different static and also dynamic experimental conditions. To the best of our knowledge, this is the first experimental work that examines the effect of using a La-modified engineered material on P recovery performance (i) under both dynamic (CSTR) and static conditions and (ii) using not only synthetic solutions but also actual P-doped wastewater. Moreover, if the lanthanum-modified magnetite (La-MM) shows an interesting ability for P recovery, its use as an eco-friendly fertilizer instead of the commercial fertilizers would definitely promote the circular economy concept and boost environmental sustainability. Practically, the objectives of this work were (i) to synthesize and deeply characterize a La-modified magnetite-based material decorated with ferrihydrite at a relatively high lanthanum–natural magnetite percentage (35%), (ii) to determine the effect of both batch and CSTR experimental conditions on P recovery efficiency from synthetic solutions, (iii) to evaluate the effect of using real wastewater instead of synthetic solutions, and (iv) to explore and better understand the involved mechanisms in the P adsorption process.

## 2. Materials and Methods

### 2.1. Feedstock Preparation

The natural feedstock was collected from a magnetite deposit located at Al-Nabaa Mountain in Al-Qabel city, Sultanate of Oman. This raw feedstock was washed several times with distilled water and dried for 24 h at 85 °C. Then, the dried material was mechanically ground into particles smaller than 0.3 mm, which were used in this study to prepare the La-modified adsorbent.

### 2.2. Preparation of Lanthanum-Modified Material

The preparation of the La-MM was carried out through according to the experimental protocol given in our previous work [21]. Briefly, 20 g of the feedstock was shaken for 72 h in 0.5 L of 1 M HCl solution. Then, this suspension was neutralized (pH = 7) by the dropwise addition of a 0.1 M NaOH solution under continuous stirring for 2 h. This acidic treatment allowed the feedstock to be coated with ferrihydrite [23]. Afterwards, La (NO_3_)_3_*6H_2_O, at a La–feedstock mass ratio of 35%, was added to the suspension and agitated for 2 h. During this agitation process, the pH was kept constant at a value of 10 through a dropwise addition of a 1 M NaOH solution to allow the formation of lanthanum oxides and their deposition on the surface of the ferrihydrite-coated feedstock. Subsequently, the suspension was centrifuged at 3000 rpm for 15 min and the solid phase was thoroughly washed with distilled water and then calcined at 200 °C for 2 h. The resulting material was labelled La-MM, kept in dry flasks, and used for both the characterization and adsorption steps.

### 2.3. Analytical Techniques Used for Materials Characterization

The La-MM was characterized using various analytical techniques such as (i) a scanning electron microscope (SEM) coupled with energy dispersive X-Ray (EDS) (Jsm-7800F, Jeol, Tokyo, Japan) for the determination of the surface morphology, (ii) a X-ray fluorescence (XRF) device (Nexqc, Rigaku, Tokyo, Japan) for the assessment of the elemental composition, (iii) a X-ray diffraction (XRD) set up (Miniflex 600, Rigaku, Tokyo, Japan) for the determination of the crystalline phases present and possible formation of La-based nanoparticles, (iv) a Micrometrics instrument (ASAP-2020, Ottawa, ON, Canada) for the evaluation of the textural properties (i.e., surface area and pore volume), and (v) a Fourier Transform Infrared (FTIR) apparatus (AlphaII, Bruker, Leiderdorp, The Netherlands) for the assessment of the surface functional groups. Additionally, the pH at the point of zero charge of the material was evaluated through the pH drift method [32]. Moreover, additional XRD, FTIR, and BET surface area measurements were carried out for the P-loaded sample to gain insights into the involved mechanisms in the P recovery process.

### 2.4. Preparation of P Synthetic Solutions and Analyses

The phosphorus synthetic solutions were prepared through dilution with distilled water of a P stock solution at a concentration of 3000 mg L^−1^. The latter solution was prepared using disodium hydrogen phosphate (Na_2_HPO_4_*6H_2_O) purchased from Sigma-Aldrich (St. Louis, MO, USA). The adjustment of the P solutions’ pH values was carried out by using either 0.1 M NaOH or 0.1 M HCl. The pH values were measured by a Mettler Toledo bench pH meter (Mettler Toledo, Columbus, OH, USA). The initial and residual P concentrations after adsorption were assessed using the Fleury method by a UV-visible spectrometer (UV-1900i, Shimadzu, Kyoto, Japan) at a wavelength of 430 nm.

### 2.5. Phosphorus Recovery in Batch Mode

The P recovery performance of the La-MM in batch mode was evaluated by agitating 0.05 g of the material in 50 mL of a synthetic solution (dose = 1 g L^−1^) at a given concentration by a magnetic stirrer containing 15 positions (Gallenkamp; Leicestershire, UK). The agitation speed was fixed to 600 rpm based on preliminary tests. During this batch work, the effect of the following parameters on the efficacy of P recovery was assessed: (i) contact times for values ranging from 1 to 1440 min, (ii) initial pH (between 3 and 11), and (iii) the initial P concentrations (from 15 to 100 mg L^−1^). These variation intervals were selected based on preliminary experiments and previous published works [21,29]. Moreover, unless specified, batch assays were realized for the following default values: a contact time of 1440 min, an initial pH of 6.2 (not adjusted), an initial P concentration of 65.0 mg L^−1^, and at room temperature (20 ± 2 °C).

The amount of P recovered in batch mode (q_B,t_ (mg g^−1^)) and the recovery yield (Y_B,t_ (%)) by the La-MM at a given time ‘t’ are calculated as follows:

(1) $$q_{B,t}=\frac{\left(C_{i}-C_{t}\right)*V}{m}$$

(2) $$R_{B,t}(\%)=\frac{\left(C_{i}-C_{t}\right)}{C_{0}}\times 100$$

where C_i_ and C_t_ (mg L^−1^) are the initial and at time ‘t’ P concentrations, respectively. M and V are the mass of the adsorbent (g) and the volume of the liquid sample (L), respectively.

Additionally, to gain better insights into the overall adsorption process, the kinetic experimental data were analyzed with classical models (i.e., pseudo-first-order (PFOM), pseudo-second-order (PSOM), and diffusion models (DM)). Similarly, the isothermal measured data were examined with three typical models (i.e., Langmuir, Freundlich, and Dubinin–Radushkevich (D-R) models). Table 1 gives the equations of these six kinetic and isotherm models and the meaning of the involved parameters.

Furthermore, the agreement between the observed and predicted kinetic and isothermal data in batch mode was assessed through the estimation of their mean absolute percentage error (MAPE_Kin_ and MAPA_Iso_) and correlation coefficients (R^2^):

(3) $$MAPE_{Kin}\;=\;\frac{\sum \left|\frac{q_{B,t,\exp}\;-\;q_{B,t,calc}}{q_{B,t,\exp}}\;\right|}{N}\;*\;100$$

(4) $$MAPE_{Iso}\;=\;\frac{\sum \left|\frac{q_{B,e,\exp}\;-\;q_{B,e,calc}}{q_{B,e,\exp}}\;\right|}{N}\;*100$$

where q_B,t,exp_ and q_B,t,calc_ and q_B,e,exp_ and q_B,e,calc_ are the observed and estimated P recovered amount at time ‘t’ and at equilibrium, respectively. N is the number of experiments runs.

All batch experiments were performed in triplicate, and the related average values are reported in the displayed figures.

### 2.6. Phosphorus Recovery in CSTR Mode

**Figure 1:**
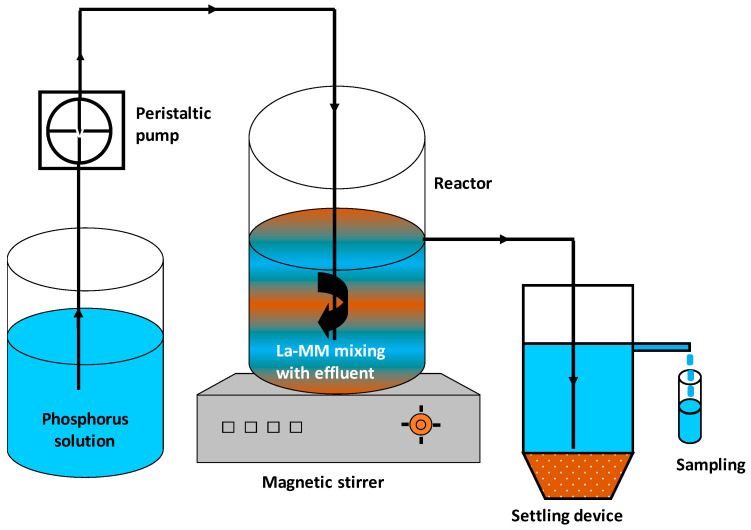
Schematic illustration of the CSTR system used for the dynamic P recovery study.

The P recovery from synthetic solutions was also studied in CSTR mode (Figure 1). This system consists of a glass reactor with a volume of 1.25 L that ensures the contact between the P ions and the La-MM particles. This reactor is connected to a 0.25 L settling device that permits the decantation of the solid adsorbent particles that have left the reactor. At the beginning of the experiment, the reactor as well as the settling setup were filled with the same solution existing in the feeding tank. Then, a given mass of the adsorbent was added to the reactor. Immediately afterward, the mixture in the reactor was shaken with a magnetic stirrer (Gallenkamp; Leicestershire, UK) and the P solution was pumped from the feeding tank to the reactor at a desired flow rate by an adjustable-flow rate peristaltic pump (Masterflex; Barrington, IL, USA). The P breakthrough curves were obtained by analyzing the P concentrations at the exit of the settling setup (Figure 1). The experiments were concluded when the P concentration at the exit of the CSTR system became identical to that of the influent for a non-negligible period.

During this work, the effect of the following parameters was evaluated: (i) initial P concentration for values of 10, 25, and 50 mg L^−1^, (ii) flow rate (from 20 to 35 mL min^−1^), and (iii) adsorbent mass (from 0.5 to 2 g). Unless otherwise stated, the initial P concentration, feeding flow rate, and adsorbent mass were fixed at 25 mg L^−1^, 28 mL min^−1^, and 1 g, respectively. These CSTR experiments were conducted at least in duplicate, and the mean values are reported in the corresponding plots.

At the end of the CSTR tests, the P recovered amount (M_P,recov_) per a given mass of adsorbent (M_La-MM_): q_e,CSTR_ (mg g^−1^) was determined through the trapezium method as below [27]:

(5) $$q_{e,CSTR}\;=\;\frac{M_{P,re\mathrm{cov},CSTR}}{M_{La-MM}}\;=\;\frac{\left[\int_{0}^{V_{tot}}C_{P,0}\;dv\;-\;\int_{0}^{V_{tot}}C_{P,t}dv\;\;\right]}{M_{La-MM}}\;=\frac{1}{M_{La-MM}}\;\left(C_{P,0}\;*V_{S}\;-\;\sum_{j=0}^{j=n}\left(\frac{C_{P,j}\;+\;C_{P,j+1}}{2}\right)\;\left(V_{j+1}\;-V_{j}\right)\right)$$

where C_P,0_ and C_P,t_ are the fixed P concentration in the feeding tank and at time ‘t’ at the outlet of the system, respectively. The C_P,j_, and C_P,j+1_ are the P measured concentrations at the exit of the system at times ‘j’ and ‘j + 1’. The ‘V_j_’ and ‘V_j+1_’ represent the collected water volume at the outlet of the CSTR system at times ‘j’ and ‘j + 1’, respectively. The V_tot_ is the full solution volume injected into the CSTR system by the peristaltic pump.

### 2.7. Phosphorus Recovery from Real Wastewater

The performance of La-MM in recovering P from a real secondary-treated effluent collected from a wastewater treatment plant (WWTP) in Muscat, Oman was assessed out in batch and CSTR modes. These experiments were conducted under the default parameters mentioned in Section 2.5 and Section 2.6. For batch experiments, the P concentration, the contact time, and the La-MM dose were fixed at 65 mg L^−1^, 24 h, and 1 g L^−1^, respectively. For CSTR assays, the P concentration, the flow rate and the adsorbent mass were equal to 25 mg L^−1^, 28 mL min^−1^, and 1 g, respectively. The collected liquid samples from these experiments were filtered through 0.22 μm PVDF filters (Whatman, Buckinghamshire, UK) and then analyzed by UV-visible at a wavelength of 430 nm.

### 2.8. Heavy Metal Release from the P-Loaded Material

It is essential to verify the ability of the P-loaded La-MM to release heavy metals especially when used in agricultural amendment. In this study, heavy metal release from both the raw feedstock and the modified material after its loading was determined through triplicate batch assays. These experiments involved stirring 1 g of the material in 1 L of distilled water for a contact time of 24 h. At the end of the agitation period, the supernatants were filtered using 0.22 μm PVDF filters, and their heavy metal contents were analyzed using an inductively coupled plasma optical emission spectroscopy (ICP-OES) (Thermo Scientific, Waltham, MA, USA).

### 2.9. Statistical Analysis

Regression analysis and plotting of the experimental and predicted data were performed with Excel 2016. Moreover, the error bars in the enclosed figures represent the standard deviation of the triplicate observed data.

## 3. Results and Discussion

### 3.1. Materials Characterization

**Figure 2:**
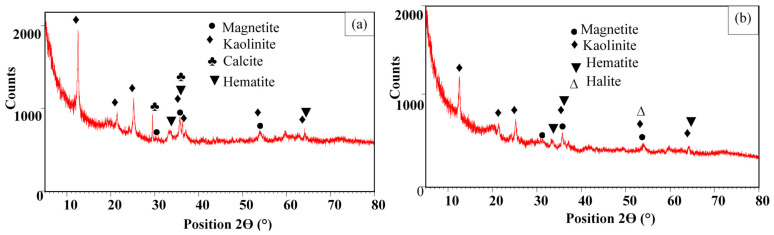
XRD spectra of the raw feedstock (**a**) and its lanthanum-modified form (**b**).

The XRD analysis of the raw feedstock shows that it is mainly composed of magnetite and kaolinite, with various peaks observed at 2θ of 30.4°, 36.3°, and 53.9°, at 64.2°, and at 12.5°, 21.4°, 25.2°, 35.7°, 36.3°, 53.9°, and 64.2°, respectively (Figure 2a) [21]. Hematite and calcite are also present but with smaller contents and are highlighted with peaks detected at 2θ of 33.3°, 35.7°, and 64.2°, at 29.5°, and at 36.3°, respectively (Figure 2a). The chemical modification with HCl and then with lanthanum nitrate induced the following changes (Figure 2b): (i) the vanishing of calcite peaks most likely due to its dissolution by the 1 M HCl solution, and (ii) the appearance of a new peak (at 2θ of 54.1°) corresponding to halite due to the high contents of Cl^-^ and Na^+^ in the suspension and calcination process (see Section 2.2). Moreover, no La-based nanoparticles were detected in the LA-MM’s XRD spectrum (Figure 2b). This indicates that lanthanum hydroxides or lanthanum oxides might be deposited as amorphous phases on the LA-MM surface. A comparable result was also found for a Chinese La-modified natural magnetite [23].

The XRD findings were in agreement with the XRF analysis (Table 2). Indeed, the chemical composition of the raw feedstock shows that besides iron, which has the largest percentage (12.4%), silicon and aluminum (which are the main constituents of kaolinite (Al_2_Si_2_O_5_(OH)_4_)) were also measured at important contents of 7.9%, and 6.5%, respectively (Table 2). In addition, the raw feedstock exhibits the presence of modest contents of Ni, Ca, Cr, and Mg. Low to negligible contents were measured for various heavy metals such as Zn, Cd, Pb, and Hg (Table 2). Besides, as expected, the P element was absent in the raw feedstock. The modification process decreased the contents of most minerals, including heavy metal elements (Table 2). However, this modification step significantly increased the O and Cl contents from 69.10% and 0.02% in the raw feedstock to 81.92%, and 5.87% in the La-MM, respectively (Table 2). The O content increase is mainly attributed to the deposition of La-based oxides on the modified material surface [33]. The Cl content increase is most probably due to feedstock pretreatment with the HCl solution (see Section 2.2). This element may be exchanged with P during the P adsorption process. These hypotheses are supported by the SEM/EDS analyses (Figure S1), which show that the raw feedstock is formed with large particles that are mainly composed of O, Fe, Mg, Al, and Si (Figure S1a). After the feedstock modification process, shiny nanoparticles could be seen at the surface of the La-MM (Figure S1b). These nanoparticles may correspond to La-(hydr)oxides [33]. The La-deposition on the modified material was also confirmed by the EDS analysis, whose spectrum shows the appearance of new La peaks with an average content of around 15.3%, suggesting that the La was partially deposited on the surface of the modified material (Figure S1b). A future quantitative analysis is, however, necessary to confirm this hypothesis. Moreover, a Cl peak was detected in the EDS spectra of the La-MM due to the raw feedstock treatment with HCl (Figure S1b).

**Figure 3:**
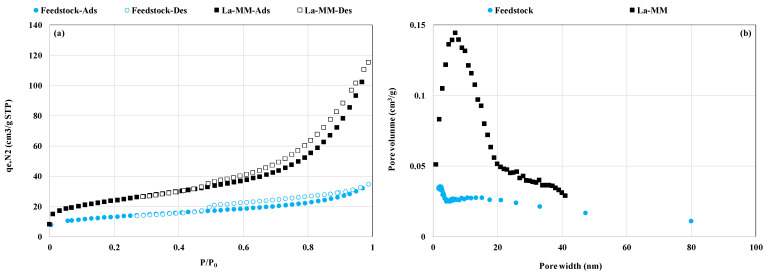
Feedstock’s and La-MM’s N_2_ adsorption and desorption isotherms (**a**) and pore size distribution (**b**) (P/P_0_: relative pressure).

In addition, the analysis of the BET results shows that the La-MM has higher BET surface area (Figure 3a) and more homogenous micropores and mesopores (Figure 3b). Indeed, the BET surface area and the total pore volume (TPV) of the raw feedstock were evaluated as 82.7 m^2^ g^−1^ and 0.160 cm^3^ g^−1^, respectively. These values were more than 86.6% and 255.5% higher than those observed for the raw feedstock, respectively (Table 2). A comparable BET surface area was measured for a Chinese La-modified magnetite [23]. It is larger than the values found for a lanthanum-modified magnetite mixed with activated attapulgite (66.9 m^2^ g^−1^) [33] and a lanthanum-modified magnetic sludge-derived biochar (7.1 m^2^ g^−1^) [34]. The improved textural properties of the La-MM can be attributed to the combined effect of (i) the coating of the raw feedstock with pure ferrihydrite after the treatment with HCl whose specific area values were estimated to 219 m^2^ g^−1^ by Liu et al. [25] and between 620 and 636 m^2^ g^−1^ by Mendez and Hiemstra [35], (ii) the formation and deposition of lanthanum-based nanoparticles on the surface of the coated feedstock with ferrihydrite, and (iii) the enlargement of material pores because of the calcination process at 200 °C (Table 2).

**Figure 4:**
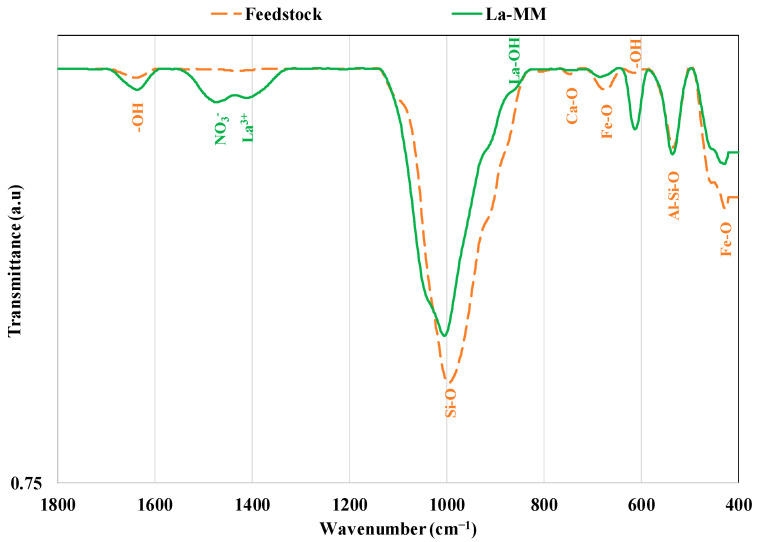
FTIR spectra of the feedstock and its La-modified form.

Concerning the surface chemistry properties, the La-MM has a slightly acidic pH at point of zero charge (pHpzc) (6.83). This suggests that for acidic pH values of the liquid effluents (lower than 6.83), the La-MM’s surface will be positively charged and consequently may exhibit high adsorption capacities of P anions through electrostatic interactions. Acidic pHpzc values have also been measured for a La-Zr-modified synthetic magnetite [16] and a La-modified natural magnetite [15]. In addition, the FTIR analysis shows that the raw feedstock is a complex material that involves different functional groups (Figure 4). Peaks observed at 427 cm^−1^, and 676 cm^−1^ correspond to Fe-O stretching vibration [17,36], and those at 534 cm^−1^ and 996 cm^−1^ are related to Al-O-Si and Si-O stretching bands from kaolinite, respectively [37,38]. Moreover, the hydroxyl stretching vibration (-OH) and -OH out-of-plan bending were detected at 1639 cm^−1^ [16,17,39] and 616 cm^−1^ [40], respectively. Finally, a small peak was observed at 746 cm^−1^ and corresponds to Ca-O band from calcite [27,41]. The feedstock modification with HCl and then with La (NO_3_)_3_ induced the following significant changes (Figure 4): (i) The disappearance of the Ca-O peak (at 746 cm^−1^) due to calcite dissolution by the HCl treatment. This observation agrees with the XRD analysis (See Figure 2b). (ii) The appearance of three peaks at 861 cm^−1^, 1411 cm^−1^, and 1471 cm^−1^ (Figure 4). The first peak (at 861 cm^−1^) corresponds to La-OH vibration [23,42], indicating that, in agreement with EDS analyses, the La was loaded at the surface of the modified material. The second peak (at 1411 cm^−1^) suggests the incorporation of La^3+^ ions into the structure of the La-MM [43], and the last one is attributed to the residual nitrates after the calcination process of the modified material with La(NO_3_)_3_*6H_2_O [43,44]. This La-MM richness would enhance the P capture efficiency from liquid effluents.

### 3.2. Batch Experimental Results

#### 3.2.1. Effect of Contact Time and Initial Aqueous pH

**Figure 5:**
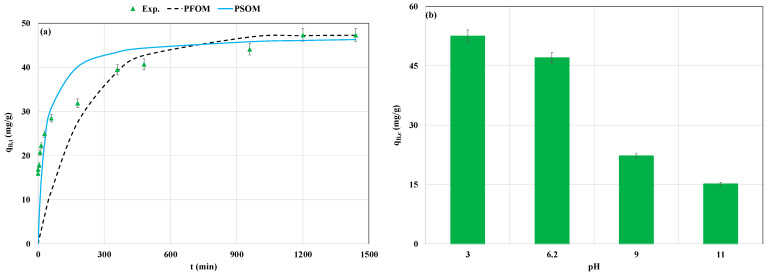
Impact of contact time duration and its fitting with kinetic models (**a**) and initial aqueous pH values (**b**) on P recovery efficiency by the La-MM (q_B,t_ and q_B,e_ are the P recovered amounts in batch mode at time t and at equilibrium).

The kinetics of P recovery by the La-MM was investigated for the experimental parameters specified in Section 2.5. Results (Figure 5a) showed that it is a remarkably time-dependent process. The P recovery process includes three different stages [32]: The first phase is observed for a duration of 1 h and possesses a relatively high kinetic rate where the P adsorbed amount reaches around 60.1% of the total adsorbed amount (Figure 5a). This is related to the P diffusion across the boundary layer around the La-MM particles. This high kinetic rate is favored by the large P concentration gradient between the aqueous phase and the adsorbent particles and the availability of huge amounts of adsorption active sites. The second phase spans from 1 h to 16 h, where the P ions continue to be recovered but at a slower rate. It is attributed to the P diffusion inside the La-MM particles. At this stage, the P concentration and the sorption active sites are significantly decreased. The last phase corresponds to an equilibrium status where these sorption sites are fully saturated and no more P adsorption is possible. The diffusion through the boundary layer seems to be the limiting phase since its corresponding diffusion coefficient (D_BL_) was around 1.6 times lower than the intraparticle diffusion coefficient (D_ITP_) (Table 3). A similar trend was observed for P recovery by a Mg/Al modified biochar [45]. The required time to reach the equilibrium was evaluated as to 20 h. It is comparable to the duration reported for a Mg-modified magnetite [18] and a La-modified magnetic sewage sludge biochar [34]. However, to reduce energetic costs related to the agitation stage under realistic conditions, a shorter contact time (i.e., 6 h) can be used. Indeed, at this time, the P recovered amount represents around 83.3% of the total adsorbed quantity (Figure 5a).

Moreover, the kinetic experimental data were well fitted with the PSO model. Indeed, the related MAPE (30.8%) was lower than that observed for the PFO model (48.0%) (Table 3). Moreover, the estimated adsorbed amount at the equilibrium shows that the PSO model is very close to the observed one (Table 3). This finding indicates that P recovery is mainly governed by chemisorption processes [46]. A similar result was reported for P recovery by a La-modified synthetic magnetite [31] and a La-modified material composed of a mixture of magnetite and attapulgite [33].

The impact of the effluent’s initial pH on P recovery by the La-MM was assessed for the experimental parameters specified in Section 2.5. Results indicate that this process is highly affected by the initial pH (Figure 5b). Indeed, the P adsorbed amount significantly decreases with the increase in the aqueous pH value. The highest P recovered amount (52.5 mg g^−1^) was observed for the lowest pH (3.0). This quantity decreased by 71.2% at an initial pH of 11 (Figure 5b). This behavior is attributed to the fact that at aqueous pH values lower than the pHpzc (6.83) (See Table 2), the adsorbent particles are mainly positively charged. This enhances the adsorption of the P anions (H_2_PO_4_^−^ or HPO_4_^2−^; pKa = 7.2) through electrostatic attraction [23]. With the increase in the aqueous pH to alkaline values, the La-MM particles become negatively charged and the main existing P anions are either HPO_4_^2−^ or PO_4_^3−^ (pKa = 12.36). Consequently, there will be a repulsion between the adsorbent particles and P anions. Moreover, the OH^−^ anions that are present in abundance in highly alkaline media may compete with P anions to be adsorbed on the available sorption sites [19]. A comparable trend was observed for P recovery by a La/Zr modified synthetic magnetite [19], a La-modified natural magnetite [23], and also a La-modified biochar [47]. For instance, increasing the pH from 4 to 10 decreased the P recovered amount by a La-modified synthetic magnetite from around 40 to less than 16 mg g^−1^ [15].

#### 3.2.2. Effect of Initial P Concentration and Competition with Foreign Anions

**Figure 6:**
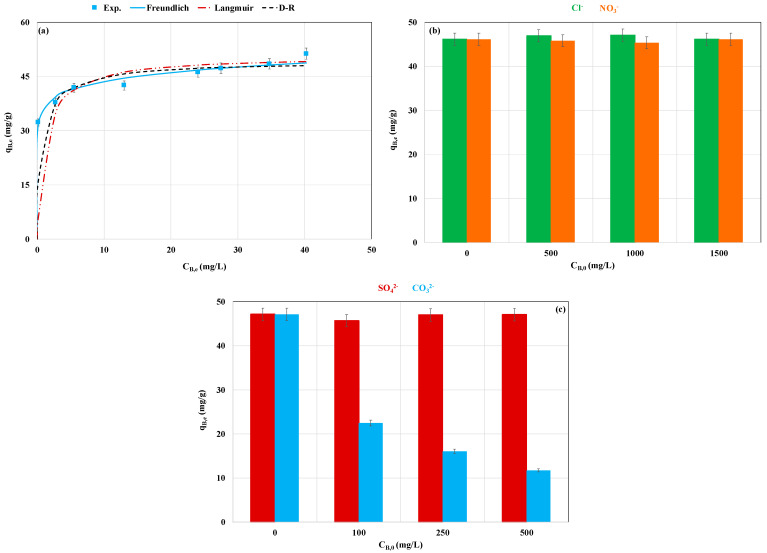
Experimental and calculated isotherm data with Langmuir, Freundlich, and D-R models of P recovery by the La-modified material (**a**) and effect of competition with chlorides and nitrates (**b**) and with sulfates and carbonates (**c**).

The adsorption isotherm study was conducted for a contact time of 24 h, an initial pH of 6.2 (without adjustment), and initial concentration in the range of 15 to 92 mg L^−1^. The La-MM was very efficient, especially at low P initial concentrations (<30 mg L^−1^) where the entire P amount was recovered. Figure 6a shows that the higher the initial concentration, the higher the P adsorbed amount. For instance, this quantity increased from 32.3 to 51.3 mg g^−1^ for initial concentrations of 32.5 and 92.0 mg L^−1^, respectively. This is attributed to the higher P concentration gradients between the effluent and the La-MM particles, which result in larger intraparticle diffusion fluxes. Moreover, the experimental data fitting with the selected isotherm models (Langmuir, Freundlich, and D-R) are given in Table 4 and Figure 6. It appears that the Freundlich model best fits the experimental data. Indeed, this model presents the highest correlation coefficient (R^2^ = 0.949) and the lowest MAPE (2.3%) (Table 4). This result shows that the P recovery by the La-MM occurs heterogeneously and in multilayers at the surface of the material [34]. Furthermore, it appears that the P adsorption is a favorable process since the estimated Freundlich constants (n) and the Langmuir constants ($R_{L}=\frac{1}{1+K_{L*\;C_{0}}}$) are lower than 1 (Table 4).

In addition, the free adsorption energy of P by La-MM was estimated by the D-R model to be 8.4 kJ mol^−1^. This value is in the interval of 8.0 to 16.0 kJ mol^−1^, suggesting that the P recovery process includes mainly chemical mechanisms. This is in accordance with the kinetic modeling findings (see Section 3.2.1). An comparable result was also reported by Lin et al. [48] during their work on P adsorption by a Zr-modified mixture of magnetite and zeolite.

The study of the effect of foreign anions on P adsorption performance shows that the co-presence of NO_3_^−^, Cl^−^ (Figure 6b), and SO_4_^2−^ (Figure 6c) did not significantly affect the recovery of P. This may be attributed to the high electron pair donor ability of P ions compared to these three anions [49]. This important selectivity towards P highlights the advantage of using this material for the real wastewater treatment. However, the presence of CO_3_^2−^ significantly reduces the P recovered amount. Indeed, reductions in adsorbed amounts of 52.3% and 75.1% were observed for CO_3_^2−^ concentrations of 100 and 500 mg L^−1^, respectively (Figure 6c). Such a behavior may be attributed to the formation of (La_2_(CO_3_)_3_) instead of LaPO_4_ [50]. Indeed, the (La_2_(CO_3_)_3_) has a much lower solubility product (ksp = 3.98 × 10^−34^) than LaPO_4_ (ksp = 3.7 × 10^−23^) [50]. Moreover, the addition of CO_3_^2−^ results in an increase in the solution pH of the solution, which is unfavorable for phosphate recovery (Figure 5b). A similar trend was observed for P recovery by various La-modified materials [33,51].

### 3.3. CSTR Experimental Results

The study of P recovery under dynamic conditions was carried out by using the CSTR system presented in Section 2.6. The effect of various parameters on P recovery performance are presented herewith.

#### 3.3.1. Impact of P Initial Concentrations

**Figure 7:**
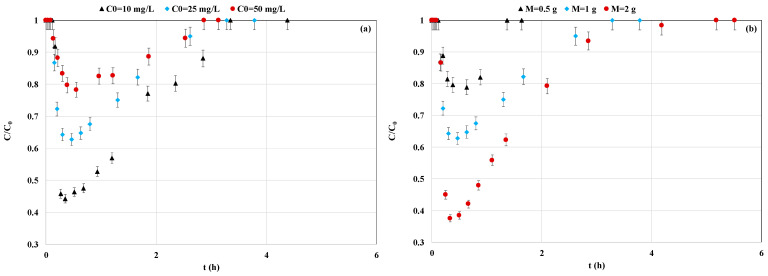

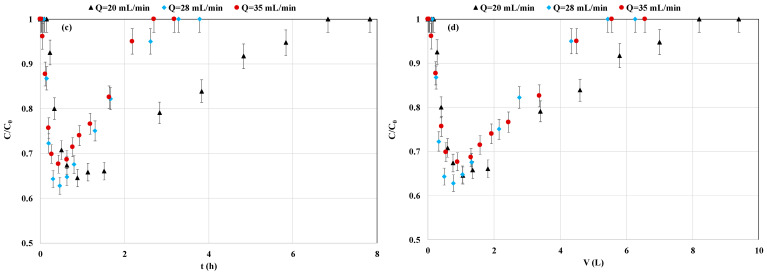
Effect of the initial concentration (**a**), adsorbent mass (**b**), and flow rate (**c**,**d**) on P recovery by the La-MM in CSTR mode.

The effect of the P initial concentration (10; 25; and 50 mg L^−1^) on its recovery by the La-MM in CSTR mode was assessed for a constant flow rate and adsorbent mass of 28 mL min^−1^ and 1 g, respectively. Results (Figure 7a) show that the P recovery is a highly time-dependent process. For instance, the relative P concentrations at the exit of the CSTR system decrease progressively vs. time until reaching a quasi-equilibrium state after 0.3 to 0.4 h. Moreover, the lower the initial P concentration is, the lower the measured P relative concentration (C/C_0_) at this plateau. As such, the lowest C/C_0_ values were assessed to be 0.55, 0.47, and 0.35 for initial P concentrations of 50, 25, and 10 mg L^−1^, respectively (Figure 7a). After this short plateau, the P is still being adsorbed by the La-MM but with a slower rate due to the increase in the C/C_0_ for all assays, until reaching a value of 1.0, corresponding to a full saturation of the adsorbent particles. At this stage, the measured concentration at the outlet of the system becomes equal to the one in the feeding tank and no more P ions can be adsorbed. As for the case of batch assays, the relatively rapid P kinetic adsorption at the beginning of the CSTR experiments is attributed to its quick diffusion through the boundary layer covering the La-MM particles. Then, the P ions slowly diffuse inside the particles’ pores, where they are retained by the active sites [27].

In addition, the P recovered amounts (q_e,CSTR_; calculated by Equation (5)) increased from 12.1 to 22.9, and then to 26.4 mg g^−1^ as the initial P increased from 10 to 25, then to 50 mg L^−1^, respectively. This trend was also observed during the batch assay and is attributed to a combination of (i) an increase in the contact probability between the P molecules and the sorption active sites of the material particles and (ii) a rise in the P concentration gradients between the effluent and the adsorbent particles [29]. A comparable trend was observed for P recovery in CSTR mode by a calcium-rich biochar [27] and a natural algal biomass [52] and in column mode by a commercial biochar [53].

#### 3.3.2. Impact of Adsorbent Mass

Three masses (0.5, 1, and 2 g) were tested for the study of P recovery efficiency in CSTR mode at constant concentration and flow rate of 25 mg L^−1^, and 28 mL min^−1^, respectively. Results (Figure 7b) show that the larger the La-MM mass, the lower the C/C_0_ plateau. For instance, for a mass of 0.5 g, the lowest measured C/C_0_ value was evaluated as 0.63. This value reaches 0.47 and 0.33 for adsorbent masses of 1 and 2 g, respectively (Figure 7b). Furthermore, the time required to reach the plateau of C/C_0_ = 1 was the shortest for the lowest mass (1.4 h). This time increases by 2.4 and more than 3.7 times for masses of 1 and 2 g, respectively (Figure 7b). For this reason, the highest recovered P amount (M_P,recov,CSTR_) was observed for an adsorbent mass of 2 g and evaluated by Equation (5) as 38.4 mg. This amount is 440.8% and 67.7% larger than those calculated for adsorbent amounts of 0.5 and 1 g, respectively. This result is primarily ascribed to the increase in the available active sorption sites which can bind with P anions present in the effluent [28]. Due to the limited number of published papers in CSTR mode, the current result analysis was extended to column assays. In this context, this behavior is in agreement with previous studies in column mode on P recovery by different materials [29,54,55]. For a constant P concentration and flow rate and of 50 mg L^−1^ and 1 mL min^−1^, Ramirez-Munoz et al. [56] proved that increasing the adsorbent bed height from 2 to 4 cm increased the P recovered mass from 22.8 to 33.0 mg, respectively.

#### 3.3.3. Impact of Flow Rate

Three assays were conducted to illustrate the effect of flow rate for values of 20, 28, and 35 mL min^−1^ and at a constant P concentration of 25 mg L^−1^ and a La-MM of 1 g. These flow rates correspond to residence times inside the reactor of 1.05, 0.75, and 0.60 h, respectively. Figure 7c illustrates the variation in the P relative concentration vs. time. It clearly shows that for the three tested flow rates, the quasi-plateau of the lowest C/C_0_ values was almost the same (between 0.62 and 0.67). However, as expected, the higher the flow rate, the faster the reaching of this plateau. Indeed, the corresponding plateau durations were evaluated as only 0.27 h for a flow rate of 35 mL min^−1^ and reached more than 0.63 h for 20 mL min^−1^ (Figure 7c). After this plateau, the P concentrations at the exit of the CSTR system start increasing due to the net reduction in the available active sorption sites. This P increase kinetic was the slowest for the lowest flow rate, and the corresponding time required to reach a C/C_0_ = 1 (adsorbent fully saturated) was equal to 6.8 h (Figure 7c). This time decreased to only 2.7 h for a flow rate of 35 mL min^−1^.

On the other hand, Figure 7d illustrates the C/C_0_ variation vs. the collected volume at the exit of the CSTR system. It shows a unique behavior for the lowest flow rate (20 mL min^−1^). Indeed, the related duration of the lowest concentration plateau was longer than the two other flow rates. Moreover, the corresponding P increase phase is the slowest and the full saturation of the media was reached after a volume of 8.2 L (Figure 7d). This volume was around 1.5 times higher than that observed for the two other flow rates. Accordingly, the calculated P adsorbed mass (q_e,CSTR_) for the lowest flow rate (by using Equation (5)) was the highest (33.8 mg g^−1^), which is around 1.5 times greater than those assessed for flow rates of 28- and 35-mL min^−1^, respectively. Such a behavior was also found for P recovery in column mode by numerous adsorbents [56,57,58]. For instance, Hamid et al. [57] showed that reducing the flow rate from 0.6 to 0.05 mL min^−1^ improved the P recovery by a flue gas desulfurization gypsum waste by 14.9%.

It is worth mentioning that no important difference exists between the P recovered quantities for flow rates of 28 and 35 mL min^−1^, with a gap of 3.2% (comparable to the experimental error) (Figure 7d). Similar results were obtained for P recovery by a Ca-rich biochar [27] and raw *Posidonia oceanica* fibers [52] for contact times varying from 2.5–4.7 h, and 0.5–1 h, respectively. This indicates that in real cases using full-scale reactors and La-MM, the P recovery efficiency may better endure flow rate variabilities than adsorbent dose and/or the P effluent concentration.

### 3.4. Effect of Using Actual Wastewater

The study of P recovery from real wastewater is an important task to carry out as it facilitates the evaluation of the impact of the existence of various dissolved minerals and organics. In the current study, the wastewater used was secondary-treated urban wastewater collected from a wastewater treatment plant in Muscat, Oman. It complies with the Omani wastewater discharging guidelines (Table S1). It has relatively low contents of anions (i.e., NO_3_^−^, Cl^−^, SO_4_^2−^). To compare P efficiency with the synthetic solutions, the wastewater P concentration was adjusted to the used default values in batch mode (65 mg L^−1^) and in CSTR mode (25 mg L^−1^). Moreover, these experiments were conducted under the other default parameters fixed in Section 2.5 and Section 2.6, respectively.

**Figure 8:**
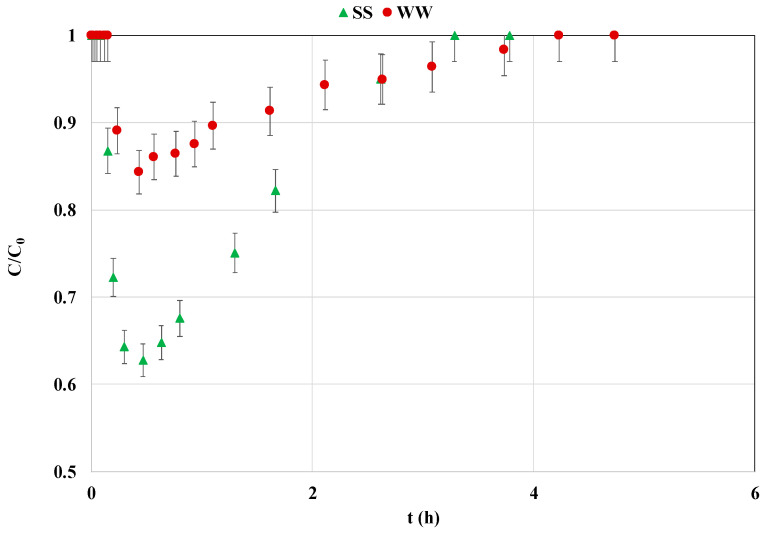
P recovery efficiency from actual wastewater and synthetic solutions in CSTR mode (SS: synthetic solution; WW: actual wastewater).

Batch experimental results indicate that substituting the synthetic solution with the actual wastewater slightly decreased the P adsorption efficiency (by 2.3%). However, in CSTR mode, the effect was much more significant (Figure 8). Indeed, the lowest measured C/C_0_ value when using the wastewater solution was evaluated to be 0.84, which is much greater than that of the synthetic solution (0.64) (Figure 8). This resulted in a net decrease in the P adsorbed mass from wastewater (q_e,CSTR =_ 10.2 mg g^−1^). This value is 2.2 times lower than that obtained for the synthetic solution (22.9 mg g^−1^). This decrease may be attributed to the presence of dissolved organic matter [33] and/or other competitive anions [59]. This effect was only observed for CSTR mode, most probably because of the corresponding low residence time (0.75 h) compared to the equilibrium required duration in batch mode (24 h). At low contact times, the existing anions in the wastewater may compete with P. However, for larger contact times, these competing anions are released in the aqueous solution from the adsorbent sorption active sites and replaced by phosphorus.

It is worth mentioning that few studies have examined the impact of using actual wastewater on P recovery by La-modified based magnetite products under batch mode. For instance, Xiao et al. [14] showed that in comparison with synthetic solutions, the P recovery by magnetite mineral microparticles from a real secondary effluent decreased by around 29%. Moreover, a removal efficiency decrease of more than 58% was reported for P removal from a water lake sample by a La(OH)_3_-modified magnetite [31]. However, some studies have shown that the use of calcium-rich wastewater may boost the P recovery by biochar [27]. Indeed, calcium ions may contribute to P precipitation as calcium-phosphate precipitates such as hydroxyapatite. Moreover, when using a La-modified porous carbon, Koilraj and Sasaki [60] found that P adsorption from actual seawater in column mode increased by 83.9% compared to synthetic solutions This finding was attributed to the presence of high concentrations of Ca and Mg ions in the environmental solution.

### 3.5. Efficiency Comparison with Other Engineered Materials

The Langmuir’s adsorption capacity of the La-MM in batch mode was evaluated to 50.7 mg g^−1^ (see Section 3.2.2). Moreover, the highest adsorbed amount in CSTR mode was equal to 33.8 mg g^−1^ for the lowest flow rate (20 mL min^−1^) (see Section 3.3.3). This decrease in P recovery efficiency is expected and usually attributed to the shorter contact times encountered in dynamic systems in comparison with the batch mode. This results in lower diffusion of P molecules from the aqueous solutions inside the pores of the adsorbent. Indeed, in the current study, the highest contact time in CSTR mode (observed for the lowest flow rate (20 mL min^−1^)) is evaluated to be 1.05 h. This duration is much lower than that used for batch assays (24 h). Nevertheless, the P recovery ability in CSTR system is relatively high and suggest that such a system can be scaled up and used for real case situations. A similar trend was observed in previous studies on P adsorption from aqueous solutions under static and dynamic conditions [24,60,61,62]. For instance, Koilraj and Sasaki [60] showed that the P adsorption capacity by a La-modified synthetic carbon decreased from 32.4 mg g^−1^ in batch mode to only 6.6 mg g^−1^ in column mode.

Table 5 summarizes the efficiency of various magnetite-based materials in recovering P in batch and CSTR modes in comparison with our La-MM. Because of the little available data on CSTR assays, we extended this comparison to laboratory columns. Moreover, it is worth mentioning that each of these assays was carried out under specific and different experimental conditions of contact time, initial concentration, adsorbent mass, etc. It is therefore difficult to get a fair comparison between all of them. However, these conditions correspond to the optimal settings giving the highest P recovered amounts. Table 5 shows that the La-MM can be considered as an effective and attractive material. Indeed, its adsorption capacity in batch mode is around 7.6, 2.9, and 1.1 times greater than the values reported for a La-modified natural vesuvianite [63], La-modified synthetic magnetite [64], and La-modified natural magnetite decorated with ferrihydrite [23], respectively (Table 5). Moreover, under dynamic conditions, the La-MM is much more efficient than various La-modified materials such as lotus seedpod derived biochar, diatomite, and synthetic porous carbon (Table 5). Other modified materials exhibited higher efficiency than the current La-MM (Table 5). This may be attributed to their much higher La [15] or Ca contents [24].

### 3.6. Heavy Metals Release

The release of heavy metals from the raw feedstock and the P-loaded La-MM was evaluated for the experimental protocol mentioned in Section 2.8. The results show that for the P-loaded La-MM, several heavy metals were not detected by the used ICP/OES device (i.e., As, and Co), while others exist with low contents, such as Pb, Ni, Mn, Cu, and Cr. (Table S2). The highest concentrations were measured for Fe (0.286 mg L^−1^) and Al (0.711 mg L^−1^). Most of these concentrations are lower than those measured for the raw feedstock (Table S2). However, to conclude about the potential use of the P-loaded La-MM as a slow-release fertilizer instead of the synthetic ones, specific pot agricultural tests are needed.

### 3.7. P Recovery Mechanisms Exploration

**Figure 9:**
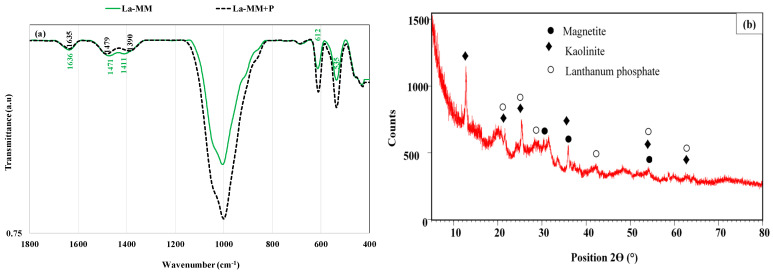
FTIR spectra of La-MM before and after P adsorption (**a**) and XRD spectrum after adsorption (**b**).

The exploration of the involved mechanisms in P recovery by the La-MM is of great importance for a successful design of the upscaling phase. Results of the kinetic (see Section 3.2.1) as well as the isotherm (see Section 3.2.2) investigations show that the P recovery includes mainly chemical mechanisms. In addition, based on the study of the pH effect (Section 3.2.1), P was found to be retained through electrostatic attraction. This latter mechanism was also reported for studies on P recovery by numerous La-modified materials [23,33,51]. The FTIR analysis of the La-MM before and after P adsorption proves that the complexation mechanism was also involved (Figure 9a). Indeed, after P adsorption, a slight decrease in the intensity of the -OH, NO_3_^−^, and incorporated La^3+^ in the modified material was observed at 1636, 1471, and 1411 cm^−1^, by around 0.18%, 0.24%, and 0.41%, respectively. Moreover, these peaks shifted by −1, +8, and −35 cm^−1^, respectively (Figure 9a). In addition, an important intensity increase in the peak observed at 611 cm^−1^ was observed (3.3%). This peak is attributed to P-O-P bending vibration [43,67] and proves the retention of P by the La-MM. The P retention through complexation mechanism was also reported for P recovery by different La-modified materials [16,31].

It is important to underline that the X ray photoelectron spectrometry (XPS) analyses of a La-modified natural magnetite [23] and a magnetite/lanthanum carbonate co-modified activated attapulgite composite [33] before and after P adsorption have shown the formation of La-O-P and Fe-O-P bonds.

Moreover, the XRD analysis of the La-MM after P adsorption (Figure 9b) indicates that the precipitation mechanism is also involved in the adsorption process. Indeed, new peaks of lanthanum phosphate (LaPO_4_) appear at 2θ of 21.6°, 25.5°, 28.6°, 42.2°, 54.2°, and 62.8° (Figure 9b). This precipitate is formed through the reaction of the dissolved La from La (hydr)oxides nanoparticles deposited at the surface of the adsorbent and the P anions contained in the feeding solution as below:

La^3+^ PO_4_^3−^ → LaPO_4_ (6)

The formation of LaPO_4_ is favored by its relatively low solubility product (3.7 × 10^−23^) as a result of the high reaction affinity between La and P [43]. The involvement of the precipitation mechanism in the recovery of P has been reported for various La-modified materials [68]. It is worth mentioning that the BET surface area and the total pore volume values of the La-MM after P recovery were evaluated to 72.7 m^2^ g^−1^ and 0.130 cm^3^ g^−1^, respectively. These values are 12.1% and 18.8% lower than those before P adsorption (see Table 2). This finding may be attributed to the formation of the LaPO_4_ precipitates, which may block some small pores. A similar trend was observed for P recovery by iron oxides [69].

## 4. Conclusions

This study shows that lanthanum modification of a natural feedstock composed of a mixture of magnetite and kaolinite produces an attractive adsorbing material with promising physicochemical properties. In batch mode, this material exhibits high potential in recovering phosphorus from aqueous synthetic solutions and real secondary-treated effluent with adsorption capacities of 50.7 and 47.3 mg g^−1^, respectively. In a continuous stirred tank reactor (dynamic conditions), the modified material keeps a relatively high ability to recover phosphorus from aqueous solutions in comparison with various engineered materials. Moreover, this efficiency tolerates some variation of flow rates more than material dose or the P effluent concentration. This recovery process is governed by various mechanisms, including mainly electrostatic attraction, complexation with different functional groups, and precipitation as a lanthanum phosphate product. The leaching of the P-loaded material shows that most of the heavy metals are either absent in the leachate or released at low concentrations. However, assessment of the agricultural valorization of this P-loaded material and its effect on both soil quality and plant growth should be undertaken in the future.

## Figures and Tables

**Table 1:** Kinetic and isotherm model equations used for the fitting of the obtained experimental data (q_B,t_: adsorbed amount at time t in batch mode; q_B,e_: adsorbed amount at equilibrium in batch mode; k_1_: kinetic recovery rate of the pseudo-first-order model, k_2_: kinetic recovery rate of the second-order model, D_f_: film diffusion coefficient; D_ip_: intraparticle diffusion coefficient; a: adsorbent’s average particle size; q_m,L_: Langmuir’s adsorption capacity; C_B,e_: equilibrium concentration in water in batch mode; K_L_: Langmuir’s coefficient; K_F_: Freundlich coefficient; n: Freundlich parameter; q_m,D-R_: adsorption capacity of D-R model; β: constant related to adsorption energy; ε: Polanyi potential.).

Name	Equation
Kinetic model	
Pseudo first-order model (PFOM)	$Ln\left(q_{B,e}-q_{B,t}\right)=Ln\left(q_{B,e}\right)-k_{1}\;t$
Pseudo second-order (PSOM)	$\frac{t}{q_{B,t}}=\frac{1}{{{k_{2}\;q}_{B,e}}^{2}}+\frac{t}{q_{B,e}}$
Boundary layer diffusion	$\frac{q_{B,t}}{q_{B,e}}=6{\left(\frac{D_{f}}{\pi a^{2}}\right)}^{1/2}\sqrt{t}$
Intraparticle diffusion	$Ln\left(1-\frac{q_{B,t}}{q_{B,e}}\right)=Ln\left(\frac{6}{\pi^{2}}\right)-\left(\frac{D_{ip}\;\pi^{2}}{a^{2}}\right)\times t$
Isotherm model	
Langmuir	$q_{B,e}=\frac{q_{m,L}\;K_{L}\;C_{B,e}}{1+K_{L}\;C_{B,e}}$
Freundlich	$q_{B,e}=K_{F}\;{C_{B,e}}^{1/n}$
Dubinin–Radushkevich (D-R)	$q_{B,e}=q_{m,D-R}\exp[-\beta \varepsilon^{2}]$

**Table 2:** Characteristics of the raw feedstock and its lanthanum-modified form (BET-SA: Brunauer–Emmett–Teller surface area; TPV: total pore volume; APS: average pore size; ND: not detected).

Material	Mineral Contents (%)															pHpzc	BET Analysis		
	O	Fe	Si	Al	Mg	Cr	Ca	Ni	Mn	Cl	Zn	Cd	P	Pb	Hg		BET-SA (m^2^ g^−1^)	TPV (cm^3^ g^−1^)	APS (nm)
Feedstock [21]	69.10	12.40	7.91	6.50	1.25	1.09	0.93	0.30	0.04	0.02	0.01	0.001	ND	ND	ND	-	44.3	0.045	6.16
La-MM	81.92	3.79	3.23	2.77	ND	1.23	0.19	0.10	0.12	5.87	0.0029	0.0005	ND	ND	ND	6.83	82.7	0.160	10.54

**Table 3:** Kinetic models parameters for phosphorus recovery by the La-MM (q_B,e,exp_ and q_B,e,calc_ are the experimental and calculated P adsorbed amounts at equilibrium in batch mode; D_BL_ and D_ITP_ are boundary layer and intraparticle diffusion coefficients).

Kinetic model	q_B,e,exp_ (mg g^−1^)	47.33
PFO model	q_B,e,calc_	47.29
	k_1_ (min^−1^)	0.0049
	R^2^	0.968
	MAPE (%)	48.0
PSO model	k_2_ (g mg^−1^ min^−1^)	0.00067
	q_B,e,calc_ (mg g ^−1^)	46.31
	R^2^	0.919
	MAPE (%)	30.8
Diffusion model	D_BL_ (×10^−13^ m^2^ s^−1^)	0.848
	R^2^	0.989
	D_ITP_ (×10^−13^ m^2^ s^−1^)	1.333
	R^2^	0.955

**Table 4:** Isotherm model parameters for phosphorus recovery by the La-modified material.

Isotherm	Parameter	Value
Freundlich	n	12.5
	K_F_	36.2
	R^2^	0.949
	MAPE (%)	2.3
Langmuir	K_L_ (L mg^−1^)	0.798
	q_m,L_ (mg g^−1^)	50.7
	R^2^	0.801
	MAPE (%)	13.9
D-R	q_m,D-R_ (mg g^−1^)	48.6
	E (kJ mol^−1^)	8.4
	R^2^	0.802
	MAPE (%)	8.9

**Table 5:** Comparison of P recovery by the La-MM with some engineered materials (C_0,B_: Initial P concentration in batch mode; D_B_: adsorbent dose in batch mode; t_B_: contact time in batch mode; T: temperature; q_m,L_: Langmuir’s adsorption capacity; C_0,D_: initial P concentration in dynamic mode; m: material mass in the column; Q: flow rate; q_e,d_: adsorbed amount at equilibrium in dynamic mode).

Adsorbent	Adsorption Experimental Conditions	q_m,L_ for Batch and q_e,d_ for Dynamic (mg g^−1^)	Reference
Batch assays			
La-modified natural vesuvianite, China	C_0-B_: 1–5 mg L^−1^; pH: 7.1; D_B_: 0.3 g L^−1^; t_B_: 40 h; T: 20 °C	6.7	[63]
La-modified synthetic magnetite	C_0, B_:1–10 mg L^−1^; pH: 7.0; D_B_: 0.1 g L^−1^; t_B_ = 2 h; T = 25 °C	13.4	[31]
La-modified synthetic magnetite	C_0,B_: 5–30 mg L^−1^; pH: 7.0; D_B_: 0.4 g L^−1^; t_B_: 24 h; T: 25 °C	17.3	[64]
La-modified synthetic porous carbon	C_0,B_: 3.1–62 mg L^−1^; pH: 7.3; D_B_: 0.5 g L^−1^; t_B_: 24 h; T: 25 °C	32.4	[60]
La-modified natural magnetite decorated with ferrihydrite, China	C_0,B_: 2–120 mg L^−1^; pH: 6.28; D_B_: 1 g L^−1^; t_B_: 24 h; T: 25 °C	44.8	[23]
La-modified synthetic magnetite and attapulgite	C_0,B_: 1–300 mg L^−1^; pH: 7.0; D_B_:1 g L^−1^; t_B_: 24 h; T: 25 °C	51.7	[33]
La-modified synthetic magnetite	C_0,B_: 0.5–250 mg L^−1^; pH: 7.0; D_B_: 0.1 g L^−1^; t_B_: 5 h; T: 23 °C	253.8	[15]
La-modified natural magnetite at a percentage of 35%, Oman	C_0,B_: 15–92 mg L^−1^; pH: natural (6.2); D_B_: 1 g L^−1^; t_B_:24 h; T: RT	50.7	This work
Dynamic assays (column or CSTR)			
La-modified commercial resin	Column mode. C_0,D_: 155 mg L^−1^; m: - g; Q: 3 mL min^−1^; T: RT	1.3	[65]
Raw algal biomass	CSTR mode. C_0,D_: 50 mg L^−1^; m: 8.3 g L^−1^; Q: 40 mL min^−1^; T: RT	3.3	[52]
La-modified lotus seedpod derived biochar	Column mode. C_0,D_: 0.5 mg L^−1^; m: 1 g; Q: 3 mL min^−1^; T: 25 °C	6.1	[50]
La-modified synthetic porous carbon	Column mode. C_0,D_: 6.2 mg L^−1^; m: 0.5 g; Q: 0.5 mL min^−1^; T: 25 °C	6.6	[60]
La-modified diatomite	Column mode. C_0,D_: 5 mg L^−1^; m: 0.2 g; Q: 0.5 mL min^−1^; T: RT	11.6	[66]
La-carbonate carbon composite	Column mode. C_0,D_: 2 mg L^−1^; m: 1 g; Q: 15 mL min^−1^; T: 25 °C	91.8	[51]
Ca-modified biochar	Column mode. C_0,D_: 50 mg L^−1^; m: 10 g; Q: 5 mL min^−1^; T: RT	66.4	[24]
	CSTR mode. C_0,D_: 50 mg L^−1^; m: 0.6 g; Q: 10 mL min^−1^; T: RT	94.9	
La-modified natural magnetite at a percentage of 35%, Oman	CSTR mode. C_0,D_: 25 mg L^−1^; m: 1 g; Q:20 mL min^−1^; T: RT	33.8	This work

## Data Availability

The raw data supporting the conclusions of this article will be made available by the authors on request.

## Supplementary Materials

The following supporting information can be downloaded at: https://www.mdpi.com/article/10.3390/ma18143383/s1, Table S1: Main physicochemical properties of the used real wastewater; Table S2: Heavy metals release ability from the raw feedstock and the P-loaded La-MM;; Figure S1: SEM/EDS analyses of the raw feedstock (a), and its lanthanum modified form (b).
