# Supplementary material for: Valorization of a Lanthanum-Modified Natural Feedstock for Phosphorus Recovery from Aqueous Solutions: Static and Dynamic Investigations
Source: Materials (Basel). 2025 Jul 18;18(14):3383. doi: 10.3390/ma18143383 (PMC12299159; doi:10.3390/ma18143383)
Supplement: Supplementary file 1 [file materials-18-03383-s001.zip › materials-3719391-supplementary.pdf]

Valorization of a Lanthanum-Modified Natural Feedstock for Phosphorus Recovery from Aqueous Solutions: Static and Dynamic Investigations

Hamed Al-Nadabi [1], Salah Jellali [1,*], Wissem Hamdi [2], Ahmed Al-Raeesi [1], Fatma Al-Muqaimi [1], Afrah Al-Tamimi [1], Ahmed Al-Sidairi [1], Ahlam Al-Hanai [1], Waleed Al-Busaidi [3], Khalifa Al-Zeidi [1], Malik Al-Wardy [1] and Mejdi Jeguirim [4]

[1] Center for Environmental Studies and Research, Sultan Qaboos University, Al-Khoud 123, Muscat P.O. Box 17, Oman; hamed@squ.edu.om (H.A.-N.); aalraeesi@squ.edu.om (A.A.-R.); fatmaalmuqimi3@gmail.com (F.A.-M.); achemtamimi@gmail.com (A.A.-T.); sidairi@squ.edu.om (A.A.-S.); aalhinai@squ.edu.om (A.A.-H.); alzeidi@squ.edu.om (K.A.-Z.); mwardy@squ.edu.om (M.A.-W.)

[2] Higher Institute of the Sciences and Techniques of Waters, University of Gabes, Gabes 6033, Tunisia; wissemhemdi@yahoo.fr

[3] College of Agricultural and Marine Sciences, Sultan Qaboos University, Al-Khoud 123, Muscat P.O. Box 17, Oman; waleedm@squ.edu.om

[4] The Institute of Materials Science of Mulhouse (IS2M), University of Haute Alsace, Centre National de la Recherche Scientifque, Unité Mixte de Recherche 7361, F-68100 Mulhouse, France; mejdi.jeguirim@uha.fr

* Correspondence: s.jelali@squ.edu.om

Supplementary materials

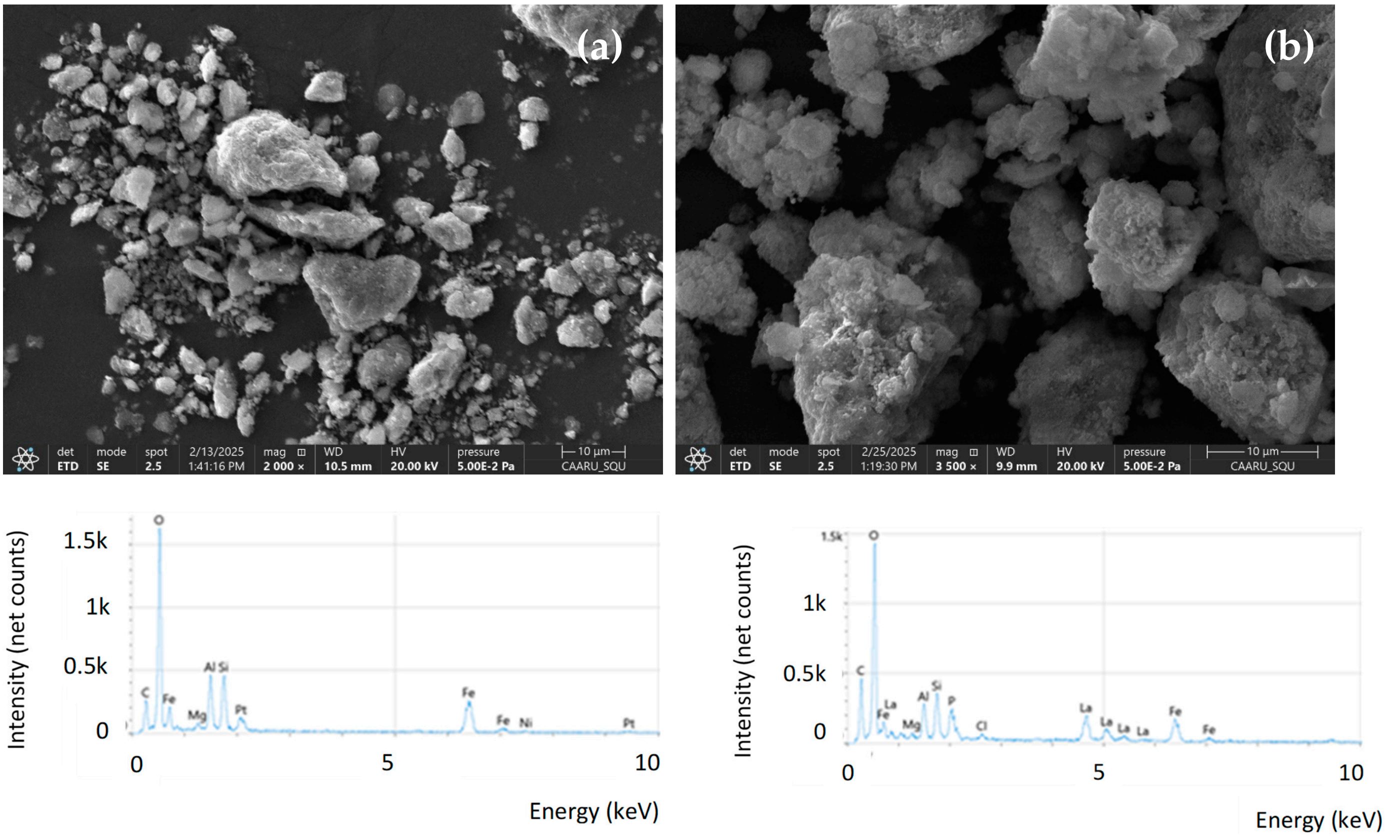

Figure S1. SEM/EDS analyses of the raw feedstock (a), and its lanthanum modified form (b)

Table S1: Main physico chemical properties of the used real wastewater

Parameter	Value
pH (-)	7.4
Electrical conductivity (mS cm^{-1})	1.4
Suspended solids (mg L^{-1})	3.5
Chlorides (mg L^{-1})	350.2
Nitrates (mg L^{-1})	5.4
Sulfates	127.2
$P\text{-}PO_4$ (mg L^{-1})	1.7
Calcium (mg L^{-1})	47.8
Sodium (mg L^{-1})	293.4
Potassium (mg L^{-1})	34.7
Magnesium (mg L^{-1})	51.2
Aluminium (mg L^{-1})	0.01

Table S2: Heavy metals release ability from the raw feedstock and the P-loaded La-MM (ND: not detected)

**Heavy metal (mg L^{-1})**	**Raw feedstock**	**P-loaded La-MM**
As	ND	ND
Mo	0.026	0.019
Zn	0.018	0.010
Pb	0.002	0.002
Ni	0.170	0.011
Co	ND	ND
Mn	0.017	0.002
Fe	4.132	0.286
Cr	0.122	0.043
Al	1.332	0.711
Cu	0.001	0.001
